# Comparative Transcriptomic Analysis Reveals Divergent Host Cell Responses to Classical and Variant Pseudorabies Virus Strains

**DOI:** 10.3390/vetsci13030226

**Published:** 2026-02-27

**Authors:** Zihan Yang, Xi Yang, Yuqing Duan, Pei Zhu, Jinping Wang, Mengting Zuo, Yun Zhou, Kewei Fan, Lei Tan, Jun Yao

**Affiliations:** 1 College of Animal Science and Technology, Yangtze University, Jingzhou 434025, China; yzh18196222349@163.com (Z.Y.); yangxi20030204@163.com (X.Y.); dyq202507@163.com (Y.D.); zy1836621633@163.com (Y.Z.); 2 Fujian Provincial Key Laboratory for Prevention and Control of Animal Infectious Diseases and Biotechnology, Longyan University, Longyan 364012, China; 3 Yunnan Tropical and Subtropical Animal Virus Diseases Laboratory, Yunnan Animal Science and Veterinary Institute, Kunming 650224, China; zpcau@sina.com (P.Z.); ynzyfeed@163.com (J.W.); 4 Hunan Provincial Key Laboratory of the TCM Agricultural Biogenomics, Changsha Medical University, Changsha 410208, China; zuomengting@aliyun.com

**Keywords:** pseudorabies virus (suid alphaherpesvirus 1), classical and variant strains, comparative transcriptomic analysis, CYP1A1 and PCNA

## Abstract

Classical and variant strains of pseudorabies virus (PRV, also called suid alphaherpesvirus 1) have been concurrently prevalent in China, with recent studies indicating that variant PRV strains may possess zoonotic potential, thereby representing a possible threat to human health. Despite this, the comparative transcriptomic responses of host cells by classical and variant PRV strains have not been thoroughly investigated. In this study, we conducted in vitro and in vivo experiments to compare the biological characteristics of the classical MinA strain and the variant WH strain. Moreover, we performed a comprehensive gene expression profiling of porcine alveolar macrophage cells (3D4/21) infected with the WH strain at 12 h post-infection, relative to those infected with the MinA strain. Our findings demonstrated that both 3D4/21 cells and Kunming mice exhibited increased susceptibility to the WH strain compared to the MinA strain. Transcriptomic analysis identified 569 significantly upregulated genes and 1091 significantly downregulated genes in WH-infected cells relative to uninfected controls, whereas MinA-infected cells showed 343 upregulated and 515 downregulated genes. Gene Ontology (GO) and Kyoto Encyclopedia of Genes and Genomes (KEGG) enrichment analyses revealed that the most prominent differentially expressed genes (DEGs) were predominantly associated with cell cycle regulation, DNA replication and immune response pathways. Notably, the genes PCNA and CYP1A1 were involved in these pathways; both of them were upregulated after PRV infection. Further investigation demonstrated that pharmacological inhibition of CYP1A1 and PCNA effectively suppressed PRV infection, suggesting their involvement in the viral infection process. Nonetheless, the precise mechanisms underlying their roles warrant further elucidation. In summary, this study provides comparative evidence for the heightened threat posed by variant PRV strains and identifies PCNA and CYP1A1 as key host–cell factors involved in the infection process, offering new potential targets for antiviral strategies against this economically significant pathogen.

## Simple Summary

The objective of this study was to compare the biological characteristics of the classical and variant pseudorabies virus (PRV) in vitro and in vivo. The results showed that the variant PRV (WH) strain exhibits enhanced infectivity in 3D4/21 cells and murine models compared to the classical MinA strain. Transcriptomic analysis revealed that infection with the WH strain induces significant alterations in host cell gene expression at 12 h post-infection, with differentially expressed genes predominantly associated with the cell cycle and immune response pathways. Notably, the genes PCNA and CYP1A1 were upregulated following infection, and pharmacological inhibition of these genes effectively attenuated PRV infection. These findings suggest that the mutated PRV strain may represent an increased threat to human health. Furthermore, the identification of PCNA and CYP1A1 as critical host factors in the viral infection process offers promising targets for the development of novel antiviral interventions.

## 1. Introduction

Pseudorabies virus (PRV), also called suid alphaherpesvirus 1, is a double-stranded linear DNA virus belonging to the subfamily *Alphaherpsvirinae* of the family *Herpesviridae* [1]. PRV is the etiological agent of pseudorabies, a significant infectious disease that severely impacts the Chinese swine industry [1]. The clinical manifestations of PRV infection primarily include severe systemic disorders and diarrhea in suckling piglets, reproductive failures in sows, impaired growth and respiratory distress in fattening pigs [2]. In addition to pigs, PRV is capable of infecting a variety of mammalian species, often causing high mortality rates [3]. Notably, recent reports from China have documented several human cases of encephalitis and endophthalmitis attributed to PRV infection, suggesting that PRV may represent an emerging public health concern [2,4].

According to their genomic characteristics, PRV strains fall into two genotypes: type I and type II [2]. Genotype II strains predominantly circulate within Chinese swine populations and encompass both classical and variant PRV strains [5]. In China, the Bartha-K61 vaccine has been effective in controlling infections caused by classical PRV strains. However, since 2021, the emergence of PRV variants in the swine industry has garnered significant attention, as the Bartha-K61 vaccine does not confer complete protection against these PRV variants [6]. In addition, these variant strains exhibit increased pathogenicity in animals, including pigs and mice, compared to the classical PRV strains [7].

RNA sequencing (RNA-seq) technology has been considered a valuable approach for elucidating the cellular mechanisms and identifying potential host factors implicated in these processes [8,9,10]. In the context of PRV, few studies have addressed the transcriptomic profiles of PK15 cells following PRV infection [11,12]. Notably, host responses to PRV infection vary across different cell lines; for instance, Wang et al. reported that PRV-GFP infection upregulated the mRNA expression levels of IFITM2 and IFITM3 genes in PK15 cells, whereas no significant changes were observed in 3D4/21 cells [13]. In the current study, the biological properties of both variant and classical PRV strains were examined through in vivo and in vitro experiments. Subsequently, a comparative transcriptomic analysis was performed on 3D4/21 cells infected with either the variant or classical PRV strain using high-throughput sequencing techniques. The findings of this study are intended to serve as a reference for elucidating the differences in biological characteristics between classical and variant PRV strains.

## 2. Materials and Methods

### 2.1. Cells, Compounds, Virus and Antibodies

3D4/21 (ATCC, CRL-2843) was maintained in our laboratory and cultured in RPMI-1640 medium supplemented with 10% fetal bovine serum (FBS) and 1% penicillin-streptomycin (P/S) at 37 °C in a humidified atmosphere containing 5% CO_2_. The variant (WH) and classical (MinA) PRV strains were isolated and preserved in our laboratory. PCNA-IA (with 98% HPLC purity, Macklin, Shanghai, China) and bergamottin (with 99% HPLC purity, Macklin, Shanghai, China) were each dissolved in dimethyl sulfoxide (Solarbo, Beijing, China) to final concentrations of 20 μM and 40 μM, respectively. These chemical agents were stored at −20 °C in the dark to ensure their stability. The antibodies targeting PRV gE were graciously provided by Professor Juan Bai of Nanjing Agricultural University, China.

### 2.2. Multiplication Kinetics of PRV WH and MinA Strains in 3D4/21 Cells

Nearly 90% confluent monolayers of 3D4/21 cells cultured in 6-well plates were infected with PRV WH or MinA strains at a multiplicity of infection (MOI) of 0.002 for a duration of two hours at 37 °C. Following the removal of the inoculum, the cells were maintained in RPMI 1640 in medium containing 2% FBS. Infectious virus was collected from both the cells and supernatants at 12, 24, 36, 48, and 60 h post-infection (hpi). Viral titers were quantified using the 50% tissue culture infectious dose (TCID_50_) assay, as described in a previous study [14].

### 2.3. Immunofluorescence Assay

Approximately 80% confluent monolayers of 3D4/21 cells seeded in 12-well plates were infected with varying MOIs (0.02, 0.05, 0.1, 0.2, 0.5 and 1.0) of PRV WH and MinA strains for a duration of two hours. Following infection, the supernatants were removed, and the cells were washed three times with PBS. Subsequently, the cells were maintained in RPMI 1640 medium supplemented with 2% FBS at 37 °C for 12 h. The cells were then fixed with 4% paraformaldehyde for 15 min, washed and permeabilized with PBS containing 0.1% Triton X-100 for 10 min. The cells were then blocked with PBS containing 3% bovine serum albumin for one hour and incubated with the primary PRV-gE antibody for 12 h at 4 °C. Following PBS rinsing, the cells were exposed to a fluorescence-labeled secondary antibody for 45 min at 37 °C in the dark. Finally, the cells were washed with PBS and examined using fluorescence microscopy.

### 2.4. Experimental Infection of Mice

A total of fifty-four female Kunming mice, aged five weeks, were procured from the Experimental Animal Center of the Three Gorges University and randomly assigned to nine experimental groups. Each group, consisting of six mice, was inoculated via hind footpad injection with varying doses (10^2^, 10^3^, 10^4^, and 10^5^ TCID_50_) of PRV WH and MinA strains, respectively. The control group received an equivalent volume of PBS. All groups were kept under identical environmental conditions, and the health status of the animals was assessed thrice daily. In addition, viral load quantification in the lungs, brain, spleen and liver of mice challenged with 10^3^ TCID_50_ of PRV strains was performed using real-time PCR, following the methodology described in a previous study [15].

### 2.5. Experiment Design, cDNA Library Construction and Sequencing

Monolayers of 3D4/21 cells at approximately 80% confluence, cultured in 6-well plates, were washed three times with PBS prior to viral infection. Subsequently, the cells were then infected with PRV WH and MinA strains at an MOI of 0.5 for a duration of 12 h. Following infection, cells from each group were rinsed with ice-cold PBS, and total RNA was isolated using Trizol reagent (Hunan Aikerui Biotechnology Co., Ltd., Changsha, China). RNA concentration and purity were then determined with a NanoDrop spectrophotometer (Thermo Fisher, Waltham, MA, USA).

Eukaryotic mRNA was enriched using Oligo (dT) magnetic beads and then fragmented into short segments employing a fragmentation buffer. The obtained mRNA fragments served as templates for first-strand cDNA synthesis using random primers. Following this, the second-strand synthesis buffer and second-strand end repair enzyme mix were introduced to the reaction system to synthesize the second-strand cDNA. The double-stranded cDNA fragments were purified with AMPure XP beads and subjected to end repair. Subsequently, sequencing adapters were ligated to the fragments using bead-based methods. Size selection of the fragments was performed again with AMPure XP beads, and the cDNA library was amplified via PCR. After completing the library construction, the library was first quantified and diluted to a concentration of 1 ng/μL. The insert size was assessed using an Agilent 2100 Bioanalyzer (Agilent Technologies, Santa Clara, CA, USA). To ensure the quality of the constructed library, RT-qPCR was conducted. Finally, the prepared library was submitted for sequencing on the Illumina HiSeq 2500 platform by a commercial company service provider (Wuhan Jinkairui Biotechnology Co., Ltd., Wuhan, China).

### 2.6. RNA Sequence Data Analysis

Adapter-contaminated, poly-N-rich, and low-quality reads were discarded. Clean reads were subsequently aligned to the reference genomes of PRV variant (KP098534) and classical strain (KX423960) and the Sus scrofa reference genome (WASHUC2.69) using the Hisat2 software (v2.2.1) [16]. For each sequenced sample, mapped reads were assembled using the StringTie software (v2.2.3) [17]. Cuffcompare software (v2.2.1) was utilized to compare the transcripts assessed in this study with the reference transcriptome, facilitating the assessment of transcriptome assembly quality and the identification of novel genes. Subsequently, CPC2 software (v1.0.1) was employed to detect new genes exhibiting coding potential. Additionally, BLAST software (v2.14.1) was applied to conduct sequence alignments against multiple databases to acquire annotation information for the putative novel genes.

Differential gene expression analysis between sample groups, each comprising three replicates, was conducted using the DESeq software (v1.48.1). Differentially expressed genes (DEGs) were identified based on the criteria of a fold change greater than or equal to 2 and an adjusted *p*-value (padj) less than 0.05, where fold change denotes the ratio of gene expression levels between the two groups. Subsequently, genes exhibiting transcriptional differences were subjected to Gene Ontology (GO) functional enrichment and Kyoto Encyclopedia of Genes and Genomes (KEGG) pathway enrichment analyses using the ClusterProfiler R package (version 4.19.2). Functional or signaling pathways were considered significantly enriched if the corrected *p*-value was below 0.05.

### 2.7. Quantitative RT-PCR

TRIzol reagent (TIANGEN, Beijing, China) was used to isolate total RNA from the cells. Approximately 1 μg of the obtained RNA was then reverse-transcribed into cDNA using a commercial kit (Promega, Madison, WI, USA). The quantitative RT-PCR (RT-qPCR) was performed to assess the relative mRNA expression levels of the target genes, using the 2^−△△CT^ method as previously described [15]. The sequences of the specific primers used for the target genes were provided in Supplementary Table S1.

### 2.8. Cytotoxicity Assay

The cytotoxicity assay was conducted in accordance with methodologies described in previous studies [15]. In brief, 3D4/21 cells were seeded in 96-well plates and treated with varying concentrations of chemical compounds or DMSO for a duration of 48 h. The CCK-8 assay was subsequently employed to measure cell viability, with the detailed procedural instructions provided in Zuo et al. [18].

### 2.9. Statistical Analysis

Each experiment was carried out at least three times independently. Statistical analysis of group data was conducted using Student’s *t*-test via GraphPad Prism 8.0 (GraphPad Software, La Jolla, CA, USA). A *p*-value of less than 0.05 was considered indicative of statistical significance, * *p* < 0.05, ** *p* < 0.01, *** *p* < 0.001, while “ns” indicates a non-significant difference.

## 3. Results

### 3.1. The Kinetics of PRV Replication in 3D4/21 Cells and Their Pathogenicity in Mice

**Figure 1:**
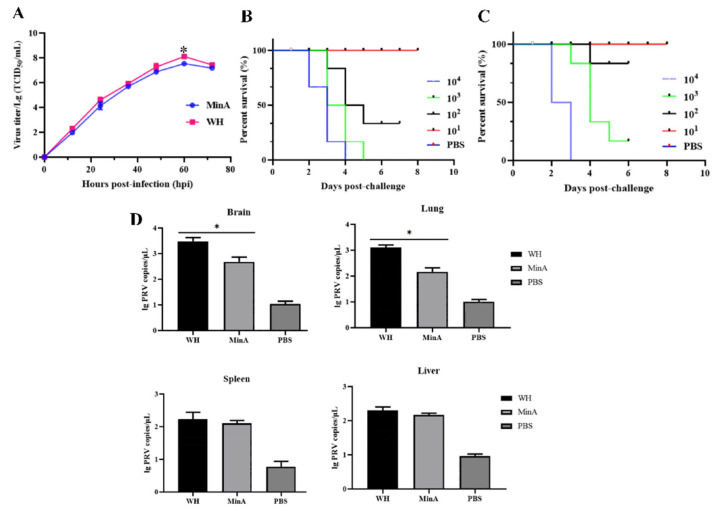
Replication dynamics of PRV in 3D4/21 cells and associated pathogenicity in mice. (**A**) One-step growth curves of the MinA and WH strains in 3D4/21 cells at an MOI of 0.01, with viral titers quantified via the TCID_50_ assay. (**B**,**C**) Kaplan–Meier survival curves analyses of mice challenged with varying viral titers of the WH (**B**) and MinA (**C**) strains. (**D**) The viral copies in different tissue samples from mice infected with the WH and MinA strains were calculated by the qPCR method. * *p* < 0.05 compared to the control group.

3D4/21 cells cultured in 6-well plates were infected with the WH and MinA strains of PRV at an MOI of 0.01. As shown in Figure 1A, both PRV strains exhibited comparable replication kinetics in 3D4/21 cells, with viral titers for the WH and MinA strains progressively increasing and reaching peak levels at 60 h post-infection, measured at 10^8.13 ± 0.21^ TCID_50_/mL and 10^7.52 ± 0.17^ TCID_50_/mL, respectively. In vivo experiments demonstrated that exposure to high doses of either PRV strain resulted in 100% mortality in mice. Specifically, administration of 10^3^ TCID_50_ and 10^2^ TCID_50_ of the WH strain led to mortality rates of 100% (6/6) and 66.67% (4/6), respectively (Figure 1B). In contrast, infection with equivalent doses of the MinA strain produced mortality rates of 83.33% (5/6) and 16.67% (1/6), respectively (Figure 1C). Moreover, at the identified challenge dose of 10^3^ TCID_50_, higher viral genome copy numbers were detected in lung and brain tissues from mice infected with the WH strain (Figure 1D).

### 3.2. RNA Sequencing Analysis

As illustrated in Supplementary Figure S1, the IFA results demonstrated that over 70% of the cells at 12 hpi exhibited the specific green (MinA) and red (WH) fluorescence targeting the PRV gE protein at an MOI of 0.5, with no evident cytopathic effects observed. However, when the MOI was 0.1, 0.05, or 0.02, the percentage of cells exhibiting corresponding specific fluorescence is comparatively low, suggesting a low rate of viral infection within the cell population. Building upon these findings, a comparative transcriptomic analysis was conducted on 3D4/21 cells infected with PRV strains WH and MinA at 12 hpi under the same MOI of 0.5. Following the removal of adapter sequences and low-quality reads, an average of 52,568,578 and 59,085,732 clean reads were obtained from the MinA and WH groups, respectively. Quality assessment revealed that the proportions of bases with Q20 and Q30 scores exceed 97.3% and 92.21% in each group, respectively (Table 1).

**Figure 2:**
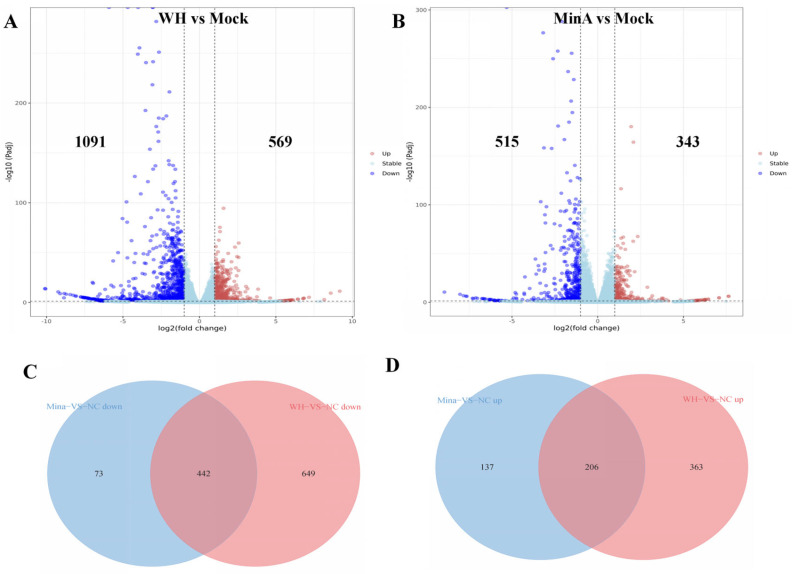
Volcano plots illustrating the differentially expressed genes (DEGs) in 3D4/21 cells infected with variant and classical strains are presented. Panels (**A**) and (**B**) displayed the DEG profiles comparing mock-infected cells to those infected with the WH strain and the MinA strain, respectively. In these plots, red and green dots corresponded to genes that were significantly downregulated and upregulated, respectively. Panels (**C**) and (**D**) showed the significantly downregulated and upregulated DEGs between sets A and B.

Compared to the control group, a total of 858 and 1660 DEGs were identified in MinA-infected and WH-infected cells, respectively. Specifically, the MinA-infected cells exhibited 515 downregulated and 434 upregulated DEGs, whereas the WH-infected cells demonstrated 1091 downregulated and 569 upregulated DEGs (Figure 2A,B). In addition, 442 DEGs were consistently downregulated and 206 DEGs were consistently upregulated across both experimental groups (Figure 2C,D).

### 3.3. GO and KEGG Analysis of DEGs

**Figure 3:**
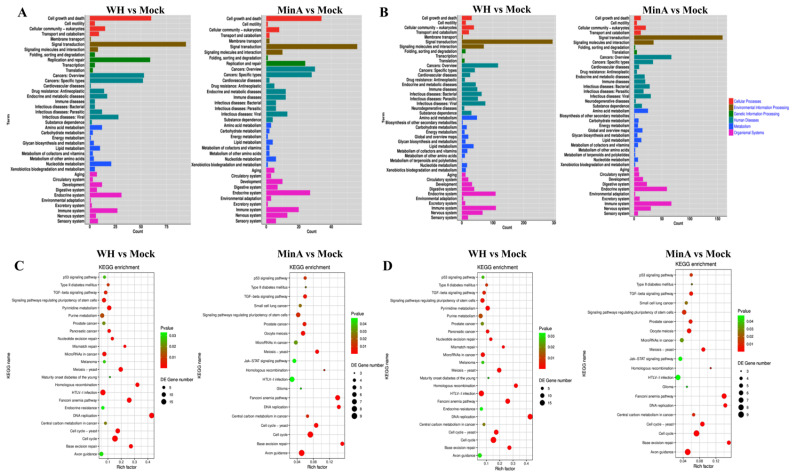
Gene Ontology (GO) classification and Kyoto Encyclopedia of Genes and Genomes (KEGG) pathway analyses were performed on 3D4/21 cells infected with either variant or classical strains of PRV. Panels (**A**) and (**C**) presented the functional characteristics of significantly downregulated DEGs between the mock-infected group and the groups infected with either WH or MinA strains by GO and KEGG enrichment analyses. Conversely, Panels (**B**) and (**D**) depict the functional characteristics of significantly upregulated DEGs between the mock-infected group and the groups infected with the WH or MinA strains, also identified via GO and KEGG enrichment analyses.

GO term enrichment analysis was performed to elucidate the biological characteristics of the DEGs, encompassing three principal functional categories, namely molecular function, cellular components, and biological processes. The analysis revealed that comparable biological events were observed in 3D4/21 cells infected with both WH and MinA strains. As shown in Figure 3A,B, the majority of significantly upregulated DEGs were predominantly associated with biological process terms such as cellular process, biological regulation, response to stimulus, multicellular organismal process, and developmental process. Within the cellular component category, these genes were mainly enriched in terms including cell, cell part, organelle, membrane, and membrane part. Regarding molecular function, the upregulated DEGs were chiefly involved in binding, catalytic activity, and molecular function regulator activities. Conversely, the downregulated DEGs were mainly enriched in BP terms such as cellular process, biological regulation, response to stimulus, metabolic process, and multicellular organismal process. In the CC category, the predominant GO terms included cell, cell part, organelle, and organelle part, while in the MF category, binding, catalytic activity, and transcriptional regulator activity were the most prominent GO terms.

Further KEGG enrichment analysis was conducted on the top 20 upregulated and downregulated pathways in 3D4/21 cells infected with the WH or MinA strain. The findings revealed minimal differences in the enrichment patterns of KEGG signaling pathways among DEGs between WH and MinA strains. Notably, pathways exhibiting significant downregulation with high enrichment scores include the TNF signaling pathway, rheumatoid arthritis, and hematopoietic cell lineage (Figure 3D). Conversely, the KEGG pathways enriched among upregulated genes predominantly involved DNA replication, the Fanconi anemia pathway, base excision repair, and the cell cycle (Figure 3C).

### 3.4. Confirmation of the Expression of DEGs by RT-qPCR

**Figure 4:**
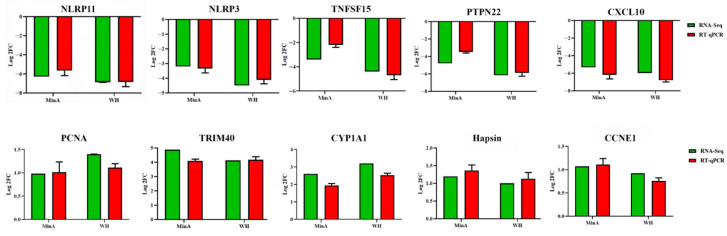
Validation of ten representative differentially expressed genes (DEGs) was performed using reverse transcription quantitative polymerase chain reaction (RT-qPCR). Specifically, 3D4/21 cells were cultured in 6-well plates and subsequently infected with either the WH or MinA strain at a multiplicity of infection (MOI) of 0.5 for a duration of 12 h. Following RNA extraction, RT-qPCR analysis was carried out to determine the relative expression levels of five upregulated and five downregulated DEGs selected for this study.

To further evaluate the accuracy and reliability of DEGs identified in PRV WH- and MinA-infected 3D4/21 cells, a total of 10 differentially expressed DEGs were randomly selected from each RNA-seq dataset and validated by RT-qPCR. As illustrated in Figure 4, the mRNA expression levels of NLR family pyrin domain-containing 11 (NLRP11), NLRP3, C-X-C motif chemokine ligand 10 (CXCL10), protein tyrosine phosphatase non-receptor type 22 (PTPN22), TNF superfamily member 15 (TNFSF15), and S100 calcium binding protein A4 (S100A4) were significantly downregulated in both WH- and MinA-infected 3D4/21 cells at 12 hpi relative to control cells. Conversely, the mRNA expression levels of tripartite motif containing 40 (TRIM40), cytochrome P450 family 1 subfamily A member 1 (CYP1A1), cyclin E1 (CCNE1), proliferating cell nuclear antigen (PCNA), and histone H3-associated protein kinase (haspin) were markedly upregulated. Overall, the findings obtained from RT-qPCR were in agreement with those derived from RNA-seq analysis. Importantly, a number of these DEGs are involved in various biological processes, such as cell cycle regulation, DNA repair mechanisms, and herpesvirus infection.

### 3.5. CYP1A1 and PCNA May Be Involved in PRV Infection

**Figure 5:**
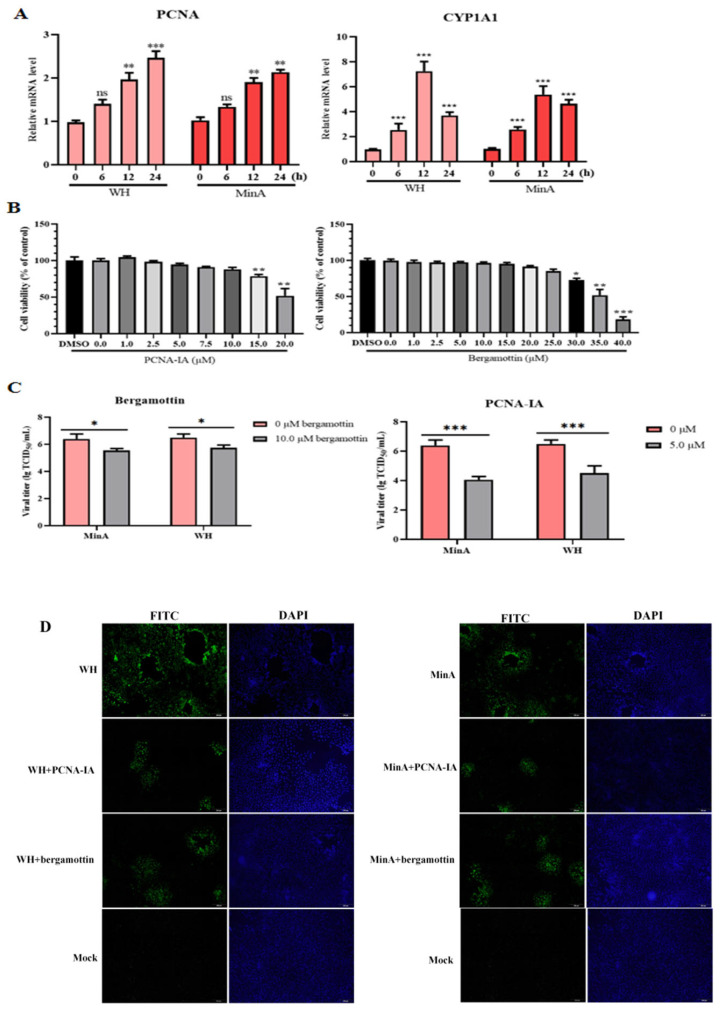
CYP1A1 and PCNA may play a role in PRV infection. (**A**) PRV infection upregulated the mRNA expression of both CYP1A1 and PCNA genes. (**B**) 3D4/21 cells were exposed to different concentrations of chemical agents for 48 h, and cell viability was determined using the CCK-8 assay (%). (**C**,**D**) 3D4/21 cells were pre-treated with PCNA (5.0 μM) or bergamottin (10 μM) for 2 h, and then exposed to WH or MinA strain at an MOI of 0.1 for 24 h. Finally, TCID50 titration (**C**) and IFA (**D**) were performed to analyze virus replication. ^ns^ *p* > 0.05, * *p* < 0.05, ** *p* < 0.01, *** *p* < 0.001 compared to the control group.

To this end, 3D4/21 cells were infected with either the WH or MinA strain of PRV for varying durations. Subsequent analyses quantified the mRNA expression levels of CYP1A1 and PCNA genes in the infected samples. Compared to the mock-infected controls, significant upregulation of CYP1A1 and PCNA mRNA was observed in cells infected with both MinA and WH strains (Figure 5A). To further elucidate the potential involvement of these genes in PRV infection. This study utilized specific chemical inhibitors: bergamottin and PCNA-IA, which selectively target CYP1A1 and PCNA, respectively [19,20]. Based on prior research findings, the cell cytotoxic effects of bergamottin and PCNA-IA at various concentrations were assessed in 3D4/21 cells following 48 h of exposure using the CCK-8 assay. As shown in Figure 5B, the half-maximal cytotoxic concentration (CC50) values for bergamottin and PCNA-IA were determined to be 34.66 ± 0.58 μM and 20.35 ± 0.44 μM, respectively. Consequently, concentrations below 20 μM for bergamottin and below 10.0 μM for PCNA-IA were selected for subsequent experiments, as these levels did not exhibit significant cytotoxic effects on 3D4/21 cells. Thereafter, the effect of these two compounds on PRV replication was evaluated through IFA and viral titer assays. The results showed that treatment with these chemical compounds significantly inhibited the infections of both WH and MinA strains (Figure 5C,D). Collectively, these results revealed the potential involvement of CYP1A1 and PCNA in PRV infection.

## 4. Discussion

PRV strains circulating in China are almost exclusively genotype II that comprises both classical and variant forms [6]. Notably, both variant and classical strains have been found to coexist in certain regions [21,22]. The findings of the present study demonstrated that infection with the variant strain at a dose of 10^2^ TCID_50_ resulted in a markedly higher mortality rate (66.67%, 4/6) than infection with the classical strain (16.67%, 1/6). Furthermore, viral copies in multiple organs of mice infected with the variant strain were more pronounced compared to those infected with the classical strain. These results suggested a heightened infectivity of the WH strain compared to the MinA strain, corroborating the results previously reported by Liu et al. [6]. Further cellular experiments indicated that 3D4/21 cells exhibited greater susceptibility to variant strains compared to classical strains. Conversely, Liu et al. reported that the classical strain (Ea) demonstrated enhanced replication capacity in PK15 cells relative to variant strains, whereas the latter one showed superior replication efficiency in SK-N-SH cells compared to the former one [6]. These observations imply potential variations in the infection mechanisms between variant and classical strains across different cell types.

The rapid advancement of RNA sequencing technology has been considered an effective method to investigate the complex host–virus interaction in vitro or in vivo [23,24]. Previous research has performed transcriptional analyses of host cells in response to PRV infection, with the majority focusing on the late stages of infection [12,25]. In the present study, we employed RNA sequencing technology to investigate transcriptomic differences between 3D4/21 infected with PRV variant and classical strains at 12 hpi. The results demonstrated that infection with the WH strain induced a greater diversity of differentially expressed genes in 3D4/21 cells relative to infection with the MinA strain. However, both PRV strains predominantly caused a significant downregulation of gene expression in 3D4/21 cells compared to the upregulation. These findings aligned with the results reported by other groups [11,26]. For instance, Shanguan et al. observed that PRV infection led to a marked reduction in the number of expressed genes in PK15 cells at 2 and 6 h post-infection, with the extent of downregulated genes substantially exceeding that of upregulated genes [11]. Similarly, Romero et al. found that PRV infection resulted in a broad suppression of cellular transcription in swine testicle cells [26]. Collectively, these findings suggest that during PRV infection, the virus suppresses host gene transcription through various mechanisms, thereby facilitating its own replication in vitro.

Subsequent analyses demonstrated that infection with the WH strain induced significant alterations in GO enrichment within 3D4/21 cells, paralleling the effects observed with the MinA strain. Notably, the PRV variant infection was associated with a greater downregulation of genes involved in host cellular immune system processes compared to infection by the classical strain. This attenuation of the cellular immune response likely facilitates viral proliferation and may partly explain the higher infection rate of 3D4/21 cells by the WH strain relative to the MinA strain. Romero et al. reported that although PRV activates the cellular NF-κB signaling pathway via DNA damage during early infection, it concurrently suppresses the transcription of downstream genes within this pathway [26]. Moreover, several PRV-encoded proteins have been shown to inhibit host innate immune responses by binding or degrading host immune-related proteins [27,28]. Given the high genomic similarity between PRV variant strains and classical strains, despite the presence of amino acid deletions, insertions, or mutations in certain encoded proteins [29], two hypotheses can be proposed regarding the differential suppression of host cell immune responses observed in this study. Firstly, the variant strains may possess one or multiple encoded proteins that exert a stronger inhibitory effect on the host immune system than those of classical strains. Secondly, the extent of host immune suppression induced by PRV infection may not be determined by viral genotype but rather by the stage of viral infection within host cells. Specifically, classical strain may predominantly inhibit host immune responses during the later phases of infection.

An analysis of KEGG pathway enrichment for DEGs in 3D4/21 cells infected with both PRV variant and classical strains revealed that the types of enriched pathways are largely consistent between the two viral infections. Importantly, infection with either variant or classical strains results in the enrichment of certain DEGs within the “DNA replication” and “cell cycle” pathways, with a greater number of genes implicated in the “DNA replication” pathway compared to the “cell cycle” pathway. During the replication of the herpes virus, the virus can exploit host proteins involved in DNA replication to facilitate its own viral replication. For instance, histone proteins have been demonstrated to be involved in the infection process of herpes viruses [30,31]. Specifically, inhibitors targeting histone acetylation modifications have been shown to markedly suppress herpes virus infection [32,33]. In addition, numerous studies have demonstrated that host proteins associated with the “cell cycle” pathway play a critical role in the replication of herpes viruses. For example, cyclin-dependent kinase 9 (CDK9) is known to regulate the cell cycle. During infection with HSV-1, its viral ICP22 protein interacted with CDK9, resulting in the inhibition of RNA polymerase II-mediated transcription [34]. Moreover, the application of specific inhibitors targeting CDK9 efficiently suppressed HSV-1 replication [34].

To validate the accuracy of the RNA-seq findings and to identify novel DEGs potentially involved in infections by both variant and classical strains of PRV, several key genes were examined. Notably, PCNA, which is localized within the nuclei of animal cells, plays a critical role in the regulation of the cell cycle and DNA replication [35]. Additionally, PCNA has been shown to facilitate HSV-1 replication through its interaction with the UL42 protein, highlighting its potential as a novel target for antiviral therapeutics [36,37]. Another gene of interest, CYP1A1, is a metabolic enzyme involved in the regulation of cancer progression [38], drug metabolism [39], and various other biological processes. Moreover, bergamottin, an inhibitor of CYP1A1, has demonstrated inhibitory effects against the infection of multiple viruses [40,41,42], while its role in herpesvirus infection remains unexplored. Our results revealed that the mRNA expression levels of these genes were significantly upregulated following PRV infection at multiple time points, corroborating the findings obtained from our RNA-seq analysis. Subsequent experiments showed that the specific inhibitors bergamottin and PCNA-IA, which target PCNA and CYP1A1 respectively, markedly inhibited the infection of both variant and classical strains of PRV in vitro. These findings suggest a potential involvement of PCNA and CYP1A1 in PRV infection, thus indicating that these genes may represent promising therapeutic targets for the treatment of PRV infection.

It is important to acknowledge several limitations present in this study. Firstly, the investigation of the biological characteristics of the two PRV strains was conducted using only a single cell line (3D/21 cells). Further research should include additional cell lines, including those derived from human sources, to provide a more comprehensive analysis. Secondly, the sample size for the titer data is limited, rendering conclusions regarding titer levels and infection kinetics preliminary and lacking robust statistical validation. Consequently, further investigation is necessary to substantiate these findings. Thirdly, the assessment of the accuracy and reliability of DEGs was performed solely through RT-qPCR assays. However, validation at the protein level was not conducted. Further efforts should address this limitation by incorporating protein-level verification. Fourthly, this study employed specific inhibitors to investigate the involvement of CYP1A1 and PCNA in PRV infection. Nonetheless, subsequent studies should incorporate gene knockdown or knockout approaches mediated by siRNA or CRISPR/Cas9 technologies to provide more comprehensive insights.

## 5. Conclusions

In conclusion, this study reveals that the WH strain exhibits a moderately greater infectivity in mice models and enhanced infectivity in 3D4/21 cell lines compared to the MinA strain. Early transcriptomic profiling indicates that both viral strains predominantly suppress host gene expression. Subsequent functional assays identified the host factors PCNA and CYP1A1 as being upregulated during infection, and pharmacological inhibition of these factors resulted in a significant reduction in viral infection in vitro. Collectively, these results elucidate critical pathogenic distinctions between PRV variants and classical strains, and suggest that PCNA and CYP1A1 represent viable host-targeted candidates for antiviral therapeutic development.

## Figures and Tables

**Table 1:** Overview of raw data generated from RNA-Seq analysis.

Sample Code	Total Reads	Clean Reads	Percentages	G + C Content	% > Q20	% > Q30
NC-1	44,863,896	44,212,762	98.61%	51.19%	97.66%	93.33%
NC-2	49,525,920	48,887,142	98.71%	50.90%	97.76%	93.55%
NC-3	48,477,180	47,852,690	98.71%	50.86%	97.86%	93.79%
MinA-1	56,578,428	55,750,596	98.54%	53.0%	97.81%	93.78%
MinA-2	47,642,964	46,935,162	98.51%	52.55%	97.63%	93.26%
MinA-3	55,797,836	55,019,978	98.61%	52.59%	97.65%	93.31%
WH-1	53,541,174	52,640,372	98.32%	60.33%	97.3%	92.77%
WH-2	51,164,538	50,196,210	98.11%	61.21%	97.05%	92.21%
WH-3	48,115,712	47,422,845	98.56%	57.99%	97.36%	92.78%

## Data Availability

The original contributions presented in this study are included in the article/Supplementary Materials. Further inquiries can be directed to the corresponding authors.

## Supplementary Materials

The following supporting information can be downloaded at https://www.mdpi.com/article/10.3390/vetsci13030226/s1, Figure S1: An indirect immunofluorescence assay was conducted to detect the gE protein in 3D4/21 cells infected with either the Ea or WH strains at 12 hours post-infection, using a multiplicity of infection (MOI) of 0.5 for each strain. Table S1: Detailed information of primers used in the present study.
