# Supplementary material for: Comparative Transcriptomic Analysis Reveals Divergent Host Cell Responses to Classical and Variant Pseudorabies Virus Strains
Source: Vet Sci. 2026 Feb 27;13(3):226. doi: 10.3390/vetsci13030226 (PMC13030817; doi:10.3390/vetsci13030226)
Supplement: Supplementary file 1 [file vetsci-13-00226-s001.zip › supplementary Table S1.pdf]

Supplementary Table S1 Detailed information of primers used in the present study

Primer name	Primer sequences	Genbank Accession No.
GAPDH-qPCR-F	AGGGCATCCTGGCTACACT	XM_008973731.2
GAPDH-qPCR-R	TCCACCACCCTGTTGCTGTA	
NLRP3-qPCR-F	CAGGCTTCTGGGACACCTT	NM_001256770.2
NLRP3-qPCR-R	TCAGAGTCCCAGGGCATAGG	
NLRP11-qPCR-F	GCCTGAATTGTGTGCCCCAT	XM_021095069.1
NLRP11-qPCR-R	CAGCAGAAGAGCAACCTCAGAT	
TNFSF15-qPCR-F	AAGGACAGGAACTTGGACCT	NM_001244555.1
TNFSF15-qPCR-R	TCATCCGGTTCTTGGTGAAGG	
CCNE1-qPCR-F	GATGGTGCTTGCAGTGAAGA	XM_005653265.2
CCNE1-qPCR-R	CGATGGCTAGAATGCACAGA	
PTPN22-qPCR-F	GTGGTCGAGGAAACTGGAGAA	XM_021090719.1
PTPN22-qPCR-R	GATCTGAGTTGGGACTCTGGA	
CXCL10-qPCR-F	CGGCACTACTGATAAGGATGG	NM_001008691.1
*CXCL1*0-qPCR-R	TGCTCAACAGCTCGGGATG	
CYP1A1-qPCR-F	CATCCGGGACATCACAGACAG	NM_214412.1
CYP1A1-qPCR-R	GCTGGGATTTGTCACCAGGTA	
haspin-qPCR-F	AAGCCTGGGATCGCTACAAC	XM_021067684.1
haspin-qPCR-R	TCGATCCCGCCAAACTCAAA	
PCNA-qPCR-F	CTGCAGATGTACCCCTTGTTGT	NM_001291925.1
PCNA-qPCR-R	CCGAAGCAGTTCTCAAAGAGC	
TRIM40-qPCR-F	AGCAGGAGAAACCGAGGTGTA	NM_001160087.1
TRIM40-qPCR-R	AAACCACCATTCTGCCACGA	
